# Supplementary material for: Causal Relationship between Aging and Anorexia Nervosa: A White-Matter-Microstructure-Mediated Mendelian Randomization Analysis
Source: Biomedicines. 2024 Aug 16;12(8):1874. doi: 10.3390/biomedicines12081874 (PMC11351342; doi:10.3390/biomedicines12081874)
Supplement: Supplementary file 1 [file biomedicines-12-01874-s001.zip › Supplementary Figure/Figure S4/Singe SNP plot of MR analysis for TL to ubm-b-1992.pdf]

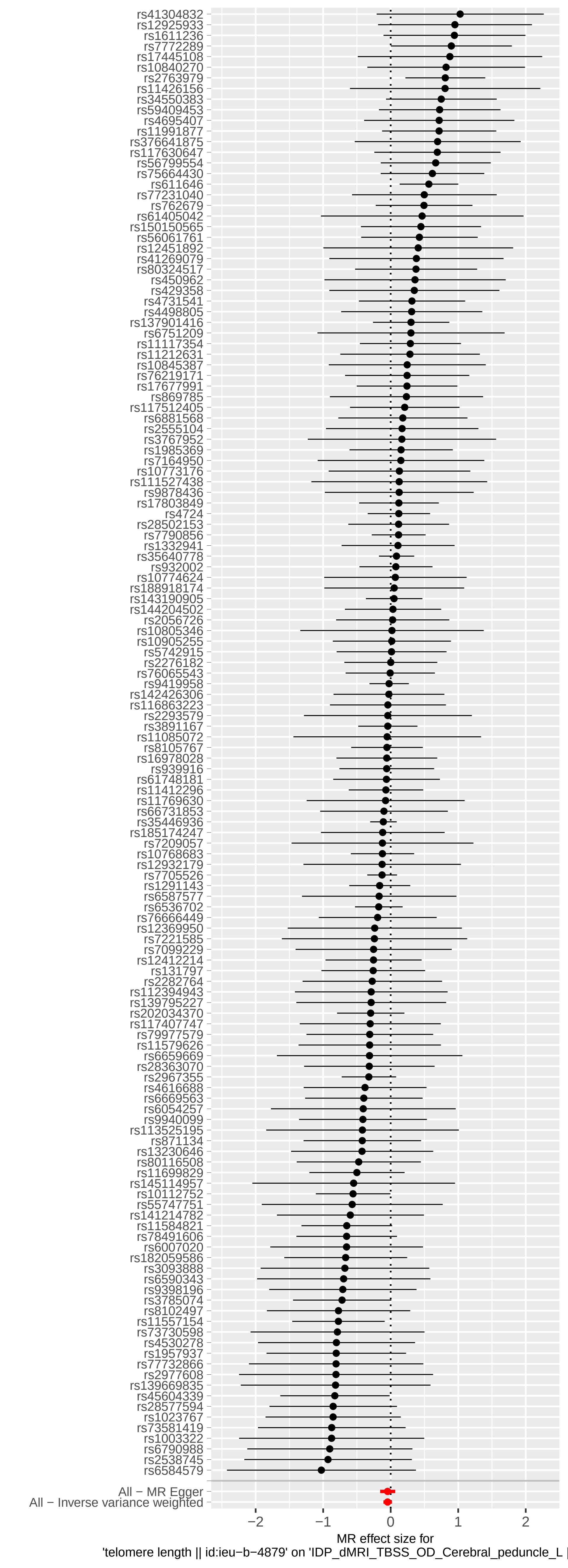
rs41304832
rs12925933
rs1611236
rs7772289
rs17445108
rs10840270
rs2763979
rs11426156
rs34550383
rs59409453
rs4695407
rs11991877
rs376641875
rs117630647
rs56799554
rs75664430
rs611646
rs77231040
rs762679
rs61405042
rs150150565
rs56061761
rs12451892
rs41269079
rs80324517
rs450962
rs429358
rs4731541
rs4498805
rs137901416
rs6751209
rs11117354
rs11212631
rs10845387
rs76219171
rs17677991
rs869785
rs117512405
rs6881568
rs2555104
rs3767952
rs1985369
rs7164950
rs10773176
rs111527438
rs9878436
rs17803849
rs4724
rs28502153
rs7790856
rs1332941
rs35640778
rs932002
rs10774624
rs188918174
rs143190905
rs144204502
rs2056726
rs10805346
rs10905255
rs5742915
rs2276182
rs76065543
rs9419958
rs142426306
rs116863223
rs2293579
rs3891167
rs11085072
rs8105767
rs16978028
rs939916
rs61748181
rs11412296
rs11769630
rs66731853
rs35446936
rs185174247
rs7209057
rs10768683
rs12932179
rs7705526
rs1291143
rs6587577
rs6536702
rs76666449
rs12369950
rs7221585
rs7099229
rs12412214
rs131797
rs2282764
rs112394943
rs139795227
rs202034370
rs117407747
rs79977579
rs11579626
rs6659669
rs28363070
rs2967355
rs4616688
rs6669563
rs6054257
rs9940099
rs113525195
rs871134
rs13230646
rs80116508
rs11699829
rs145114957
rs10112752
rs55747751
rs141214782
rs11584821
rs78491606
rs6007020
rs182059586
rs3093888
rs6590343
rs9398196
rs3785074
rs8102497
rs11557154
rs73730598
rs4530278
rs1957937
rs77732866
rs2977608
rs139669835
rs45604339
rs28577594
rs1023767
rs73581419
rs1003322
rs6790988
rs2538745
rs6584579
All − MR Egger
All − Inverse variance weighted
−2
−1
0
1
2
MR effect size for
'telomere length || id:ieu−b−4879' on 'IDP_dMRI_TBSS_OD_Cerebral_peduncle_L |
